# Non‐invasive optical biopsy by multiphoton microscopy identifies the live morphology of common melanocytic nevi

**DOI:** 10.1111/pcmr.12902

**Published:** 2020-06-17

**Authors:** Griffin Lentsch, Manuel Valdebran, Inga Saknite, Janellen Smith, Kenneth G. Linden, Karsten König, Ronald J. Barr, Ronald M. Harris, Bruce J. Tromberg, Anand K. Ganesan, Christopher B. Zachary, Kristen M. Kelly, Mihaela Balu

**Affiliations:** ^1^ Beckman Laser Institute and Medical Clinic University of California Irvine CA USA; ^2^ Department of Dermatology University of California Irvine CA USA; ^3^ Department of Biophotonics and Laser Technology Saarland University Saarbrucken Germany; ^4^ JenLab GmbH Jena Germany; ^5^ Present address: Vanderbilt Dermatology Translational Research Clinic Department of Dermatology Vanderbilt University Medical Center Nashville TN USA; ^6^ Present address: National Institute of Biomedical Imaging and Bioengineering National Institutes of Health Bethesda MD USA

**Keywords:** autofluorescence imaging, melanocytic nevi, melanoma, multiphoton excitation microscopy, optical microscopy, pigmented moles, pigmented nevi

## Abstract

Multiphoton microscopy (MPM) is a promising non‐invasive imaging tool for discriminating benign nevi from melanoma. In this study, we establish a MPM morphologic catalogue of common nevi, information that will be critical in devising strategies to distinguish them from nevi that are evolving to melanoma that may present with more subtle signs of malignancy. Thirty common melanocytic nevi were imaged in vivo using MPM. Quantitative parameters that can distinguish between different types of nevi were developed and confirmed by examining the histology of eleven of the imaged nevi. MPM features of nevi examined included cytologic morphology of melanocytes in the epidermis and dermis, the size and distribution of nevomelanocytes both within and around nests, the size of rete ridges, and the presence of immune cells in the dermis. Distinguishing features include cytological morphology, the size of nevomelanocytes, the size of nevomelanocyte nests, and the distribution of nevomelanocytes. Notably, these distinguishing characteristics were not easily appreciated in fixed tissues, highlighting essential differences in the morphology of live skin. Taken together, this work provides a morphologic compendium of normal nevi, information that will be critical in future studies directed at identifying melanocytic nevi that are evolving to melanoma.

**Significance**

Successful translation of multiphoton microscopy to the clinical setting requires a good understanding of the live skin tissue morphology, which may differ significantly from the processed tissue morphology. This study establishes a MPM morphologic catalogue of common nevi, information that will be critical in devising strategies to distinguish them from nevi that are evolving to melanoma. An immune response in common melanocytic nevi is identified and confirmed histologically for the first time. This study proposes several quantitative parameters based on MPM features of common nevi that can distinguish between different types of nevi.

## 1 INTRODUCTION

Common melanocytic nevi, also known as “moles,” are benign growths of melanocytes. Most nevi are benign, but some can be difficult to distinguish from early‐stage melanoma leading to unnecessary excisions (H. K. Koh et al., 1996; Osborne, Chave, & Hutchinson, 2003). Multiphoton microscopy (MPM) is a laser scanning imaging technique that can provide label‐free contrast through optical signals generated by non‐linear near‐infrared laser interactions with tissue molecular components. Clinical MPM is a recent and rapidly developing field of research in dermatology, holding great promise as a non‐invasive imaging tool in clinical practice, particularly for early diagnosis. Our group and others have demonstrated the potential of MPM for non‐invasive imaging of pigmented lesions and detection of melanoma (Arginelli et al., 2013; Balu et al., 2014; Dimitrow et al., 2009; Seidenari et al., 2013). However, these studies include limited information about the MPM morphology and cytology of common nevi and no histology confirmation of the identified MPM features. Knowledge about the in vivo morphology of benign melanocytic nevi, which may differ significantly from the processed tissue morphology, is necessary for the differentiation of benign melanocytic nevi from early malignant melanomas.

In this study, we seek to explore the range of in vivo morphologies of different types of common melanocytic nevi in human skin in order to identify key MPM features of these skin lesions. Melanocytic nevi are composed of nevus cells, which differ from normal melanocytes as they are arranged in clusters or “nests” and by rarely if ever showing dendritic processes. (Lever & Schaumburg‐Lever, 1990) Nevus cells lie in nests in the lower epidermis in junctional nevi and mainly in the dermis of intradermal nevi. (Lever & Schaumburg‐Lever, 1990) Compound nevi possess features of both junctional and intradermal nevi.

We assess qualitatively, by comparison with histology, the presence of nests of nevomelanocytes and their cytological features in the in vivo MPM images. We also identify and confirm histologically, for the first time, the immune response in some of these melanocytic lesions. We propose several quantitative parameters related to the size of nevomelanocytes, the size of the nests, and the percentage of nevomelanocytes located outside the nests. We show that these metrics can distinguish between different types of nevi and may be relevant in assessing nevi in evolution or in distinguishing benign nevi from early‐stage melanoma, or in quantifying an anti‐melanocyte immune response induced by checkpoint inhibitors.

## 2 MATERIALS AND METHODS

### 2.1 Clinical multiphoton tomograph

We used an MPM‐based clinical tomograph (MPTflex, JenLab, GmbH, Germany) for the in vivo imaging of the pigmented lesions in human skin. This imaging system consists of a femtosecond laser (Mai Tai Ti:Sapphire oscillator, sub‐100 fs, 80 MHz, tunable 690–1020 nm; Spectra‐Physics), an articulated arm with near‐infrared optics, and beam scanning module. The imaging head includes two photomultiplier tube detectors used for parallel acquisition of two‐photon excited fluorescence (TPEF) and second harmonic generation (SHG) signals. The excitation wavelength used in this study was 790 nm. The TPEF signal was detected over the spectral range of 410 to 650 nm, whereas the SHG signal was detected over a narrow spectral bandwidth (385–405 nm). We used a Zeiss objective (40×, 1.3 numerical aperture, oil immersion) for focusing the laser light into the tissue. The laser power used was 5 mW at the surface of the skin and up to 30 mW deeper into the superficial dermis of the skin. We acquired the MPM data as z‐stacks of en‐face images from the stratum corneum to the superficial dermis. Typically, the field of view (FOV) for each optical section was about 200 × 200 μm^2^ and the step between the optical sections 5 μm. Due to the limited FOV of each individual scan, in order to sample a larger area of the lesion, we acquired several stacks of images within each lesion. Thus, a total of 2,462 images were acquired for this study, corresponding to an average of 82 images for each nevus. Images were 512 × 512 pixels and were acquired at approximately 6 s per frame. For several lesions, we acquired vertical cross‐sectional, “histology‐like” images from the stratum corneum to superficial dermis. These images were 1,024 × 1,024 pixels and were acquired at approximately 30 s per frame. All images were color‐coded such that green and blue represent the TPEF and SHG signals, respectively. In MPM imaging of skin, the contrast mechanism is based on two‐photon excited fluorescence (TPEF) signal from NADH, FAD, keratin, melanin, and elastin fibers and on second harmonic generation (SHG) signal from collagen.

### 2.2 Study design

#### 2.2.1 Patients

Thirty pigmented lesions in twenty‐eight patients were imaged in vivo by MPM. In order to address inter‐observer variability, all lesions that were not biopsied (19) were diagnosed as common nevi by three independent dermatologists. The pigmented lesions with a clinically suspicious appearance (11) were biopsied following MPM imaging. The histological diagnosis was performed by three independent dermatopathologists. The topographic location and pathologic characteristics along with MPM features corresponding to each case are summarized in Table 1.

**Table 1:** Patient age, location of the lesion, clinical diagnosis, MPM morphological features, and pathologic diagnosis of nevi

Case no	Age (years)/Gender	Location	Clinical diagnosis	MPM features			Pathology diagnosis
				Nests of nevus cells	Elongated rete ridges	Immune response	
1	57/M	Back	JN	No	Yes	No	x
2	34/F	Leg	CN	Yes	No	No	x
3	28/F	Arm	JN	Yes	No	No	x
4	22/F	Arm	JN	Yes	No	No	x
5 (Fig. S3)	22/F	Arm	JN	Yes	No	Yes	x
6 (Figure 1)	25/F	Arm	JN	Yes	Yes	No	x
7	63/F	Abdomen	JN	Yes	No	No	x
8	24/M	Arm	CN	Yes	No	No	x
9	20/F	Arm	CN	Yes	No	No	x
10	23/F	Arm	JN	No	Yes	No	x
11	33/F	Arm	CN	Yes	No	No	x
12	57/F	Leg	JN	No	Yes	No	x
13	57/F	Arm	JN	No	Yes	No	x
14 (Fig. S2)	55/M	Chest	IDN	Yes	No	No	x
15	23/M	Arm	JN	Yes	No	Yes	x
16	57/F	Back	IDN	Yes	No	No	x
17	48/F	Leg	CN	Yes	No	No	x
18	89/M	Leg	JN	No	Yes	No	x
19	28/M	Arm	IDN	Yes	No	No	x
20	42/M	Arm	AN	No	No	Yes	IDN
21	47/F	Arm	IN versus Melanoma	Yes	No	No	IDN
22 (Figure 2b)	67/F	Abdomen	AN	Yes	No	No	IDN
23 (Figure 6)	27/M	Back	AN	No	No	Yes	IDN
24	56/F	Back	Nevus versus BCC	No	No	Yes	IDN
25 (Figure 2a, S1)	23/F	Abdomen	IDN	Yes	No	Yes	CN/IDN
26	46/F	Back	AN	Yes	No	Yes	CN
27 (Figure 5)	27/F	Arm	RN‐CN	No	No	Yes	RN‐CN
28	89/M	Arm	RN‐IDN versus Melanoma	No	No	No	RN‐IDN
29	52/F	Back	AN versus melanoma	Yes	Yes	No	CN
30	46/M	Abdomen	AN	No	Yes	Yes	CN

Note

Abbreviations: AN, atypical nevus; BCC, basal cell carcinoma; CN, compound nevus; IDN, intradermal nevus; IN, irritated nevus; JN, junctional nevus; RN, recurrent nevus.

All in vivo measurements were conducted according to an approved institutional review board protocol of the University of California, Irvine (HS No. 2011‐8494), with written informed consent obtained from all patients.

#### 2.2.2 Image analysis

We used ImageJ for both the qualitative and quantitative analyses. The qualitative analysis included: (i) basic processing tasks of the MPM images for enhancing contrast and highlighting features of interest; and (ii) comparison of the MPM and histologic images of biopsied nevi in order to identify and correlate their morphological features.

The quantitative analysis included measurements of: (1) nevomelanocyte sizes—We calculated the average cell diameter using a semi‐automatic segmentation method within ImageJ. We used the following procedure for nevomelanocyte segmentation: Gaussian blur (sigma: 2.0), manual outline selection of cell(s) to be measured, signal thresholding based on signal in outlined area, image binarization, erosion and/or dilation (if necessary to outline complete cell), watershed (if necessary to distinguish neighboring cells), and Feret's diameter measurement for each segmented cell. We used a two‐tailed *t* test to determine whether the nevomelanocyte sizes in the junctional and compound nevi were significantly different from size of nevomelanocytes in the nests of intradermal nevi. We combined the results from junctional and compound nevi into one group since, in compound nevi, MPM captured mostly the epidermal component of the nevus, namely the epidermal nests; (2) nevomelanocyte nest sizes—We measured the major axis of each nest at its maximum imaged size as determined from the MPM cross‐sectional en‐face images. The average size of each nest was calculated based on two independent measurements. We used a two‐tailed *t* test to determine whether the nevomelanocyte nest sizes: (i) were significantly different in the two groups of junctional/compound and intradermal nevi; and (ii) significantly changed within the imaging depth; and (3) the percentage of nevomelanocytes outside the nests—For each en‐face image acquired within the stack, we measured the number of cells inside the nests through the ratio of the nest area to the average area of the melanocytes within the nest. We counted manually the sparse melanocytes outside nests and calculated their percentage by dividing their number to the total number of nevomelanocytes located within nests for each imaging volume.

## 3 RESULTS

We analyzed both qualitatively and quantitatively the MPM images acquired in vivo in thirty common nevi in human skin (see Table 1 for a summary of the lesion location and diagnosis). Besides the enhanced pigmentation of keratinocytes, which may also be present and imaged in pigmented normal skin (Balu et al., 2014; Saager et al., 2015), the MPM features of nevi included: (i) nests of nevomelanocytes; (ii) elongated rete ridge epidermal extensions into the underlying connective tissue; and (iii) an immune response in the form of melanophages and inflammatory cells in the dermis. These features are discussed below.

### 3.1 Nests of nevomelanocytes

**Figure 1:**
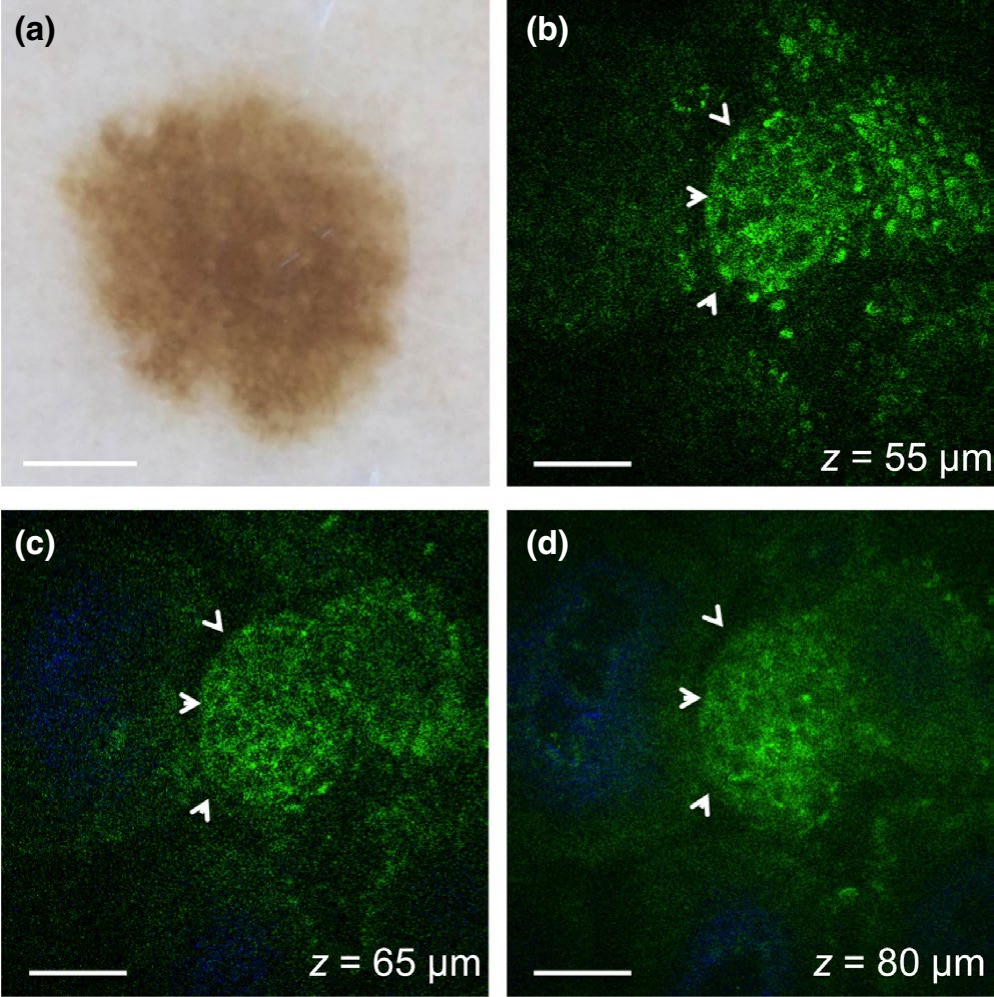
In vivo MPM of nevomelanocytes in a junctional nevus. (a) Clinical image (Dermlite FOTO, Dermlite, Inc), Case 6. Scale bar is 1 mm. (b–d) MPM images of the nevus in (a) showing a nest of nevomelanocytes (arrows) at different depths surrounded by pigmented keratinocytes (green, visualized through bright fluorescence from melanin) (b) and dermal papillae (blue) collagen (c,d). Scale bar is 40 μm

**Figure 2:**
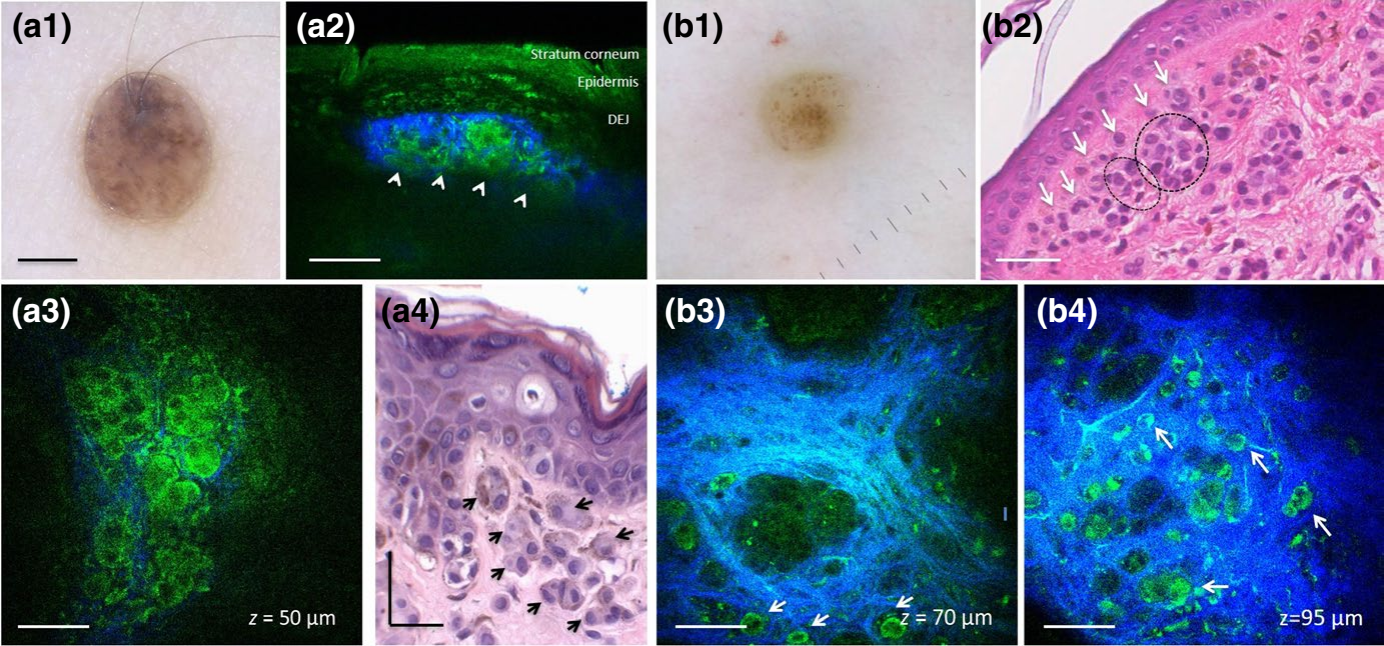
In vivo MPM of nevomelanocytes in intradermal nevi. (a1) Clinical image (Dermlite FOTO, Dermlite, Inc), Case 25. Scale bar is 2 mm. (a2, a3) Vertical cross‐sectional (a2) and en‐face (a3) MPM images of the nevus in (a1) showing a nest (arrows) of epithelioid nevomelanocytes displaying rounded large nuclei and surrounded by collagen (blue) in the papillary dermis. (a4) Hematoxylin and eosin (H&E) histological section of the lesion in (a1) showing nests of epithelioid nevomelanocytes in the papillary dermis; (b1) Clinical image (Dermlite FOTO, Dermlite, Inc), Case 22. (b2) H&E histological section of the lesion in (b1) showing sparse melanocytes (arrows) around a nest of nevomelanocytes in the papillary dermis (circle); scale bar is 40 μm. (b3, b4) En‐face MPM images of the nevus in (b1) showing sparse nevomelanocytes (arrows) around nests of nevomelanocytes surrounded by collagen (blue) in the papillary dermis at depths of 70 μm (b3) and 95 μm (b4). Scale bar is 40 μm in all MPM images

The presence or absence of nests was consistent in all the stacks acquired in 60% of the patients. In the rest of the patients, the nests of nevus cells were imaged on average in 49% of the total number of stacks acquired. The different cytological structures of the nevomelanocyte nests can be visualized in the representative MPM images from a junctional nevus in Figure 1 and from two intradermal nevi in Figure 2. In Figure 1 (Case 6), the nest imaged in the junctional nevus is located at the dermo‐epidermal junction, clearly attached to the epidermis based on its close proximity to the basal cells (pigmented cells visualized through bright fluorescence from melanin, Figure 1b).

Figure 2 (a1–a4) shows nevomelanocytes arranged in nests in the papillary dermis of an intradermal nevus imaged by MPM (Case 25 in Table 1). Figure 2 (b1–b4) includes a representative example of MPM images that show sparse nevomelanocytes located outside the nests of an intradermal nevus (Case 22). The cytological appearance of nevomelanocytes was consistent in all predominantly intradermal nevi and correlated well with histology. In an effort to provide a side‐by‐side correlation of the MPM and histology images of nevomelanocytes, we imaged ex vivo by MPM unstained tissue sections (obtained from the remaining paraffin blocks) of the biopsied lesions included in this study. We compared the MPM images with histologic (H&E‐stained) images of adjacent tissue sections. Figure S1 illustrates an example of such images acquired from the same nevus shown in Figure 2 (a1–a4, Case 25). Although the same clusters of nevomelanocytes can be visualized in both MPM and corresponding histologic images, the MPM images clearly show the difference between the nevomelanocyte appearance in the processed tissue (Figure S1) and their live morphology captured by MPM in vivo (Figure 2 a3).

#### 3.1.1 Size of the nevomelanocyte nests

**Figure 3:**
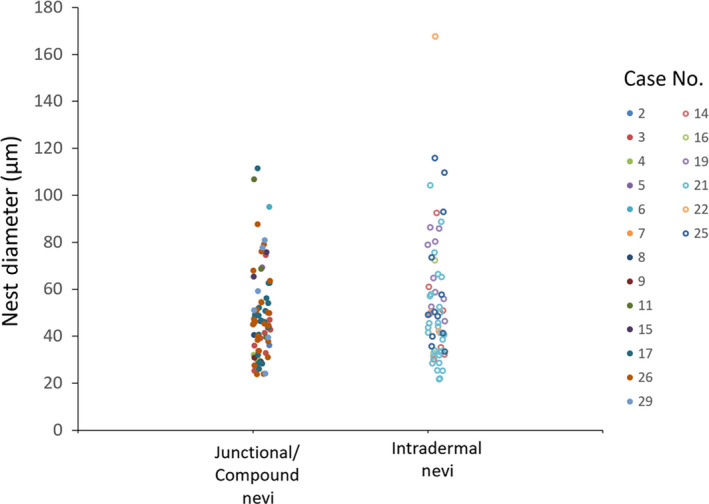
Distribution of nevomelanocyte nest diameters in junctional/compound and intradermal nevi. Data represent individual nest diameter measurements for each lesion. Case numbers correspond to those listed in Table 1

There was no significant difference between the size of nevomelanocyte nests in the two groups of junctional/compound and intradermal nevi. In all nevi, the size of the vast majority of the nests (~90%) was between 20 and 80 μm and did not significantly change with the imaging depth. Nine of the thirteen largest nests were located in intradermal nevi and had sizes within the range of 85–170 μm. This analysis included 78 nests visualized in 13 junctional/compound nevi and 62 nests in 6 dermal nevi. Figure 3 shows the distribution of the nevomelanocyte nest diameters measured in the two groups of nevi (junctional/compound versus dermal) for different patients.

#### 3.1.2 Size of nevomelanocytes

**Figure 4:**
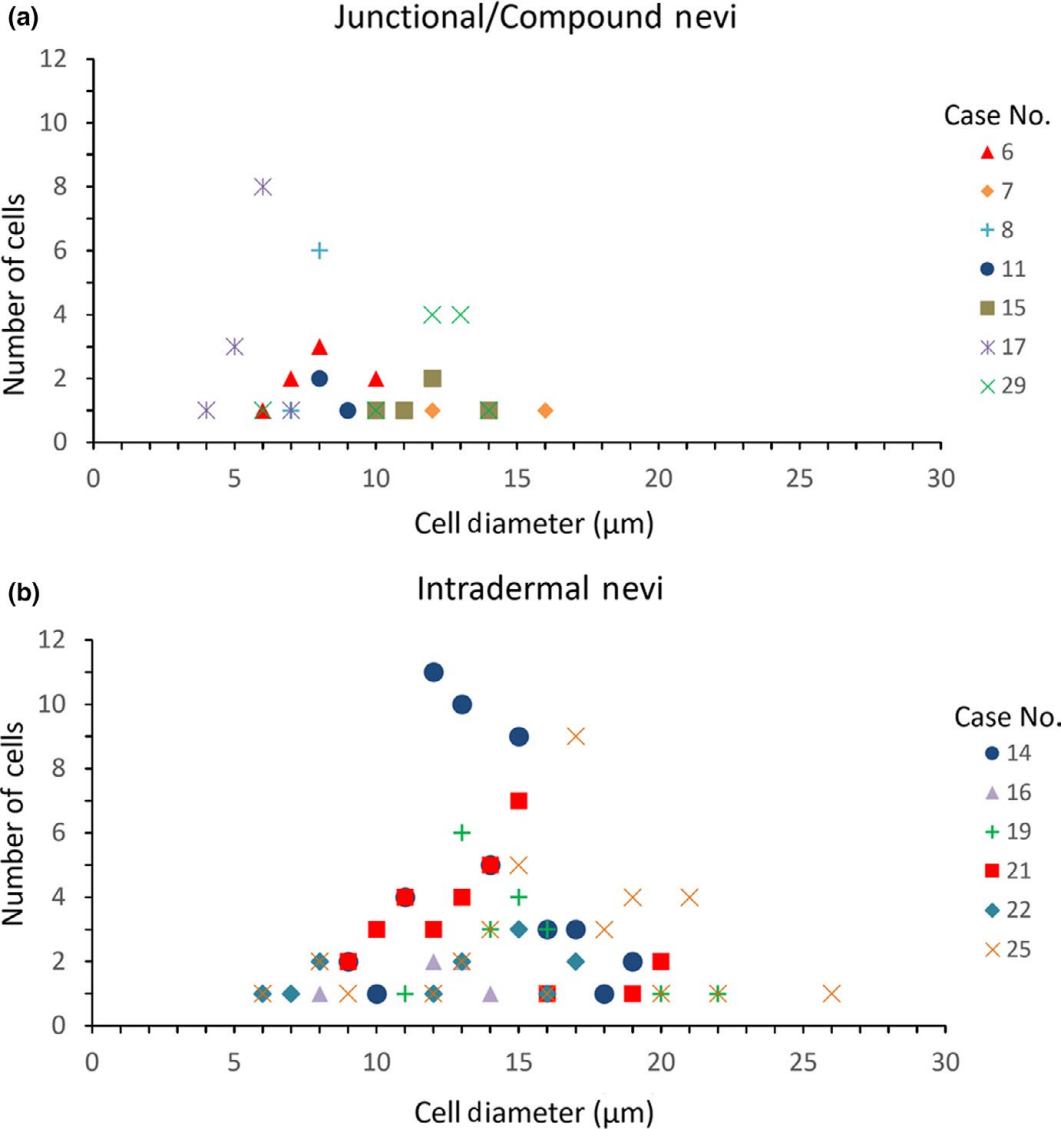
Distribution of nevomelanocyte cell diameters in junctional/compound and dermal nevi. Data represent number of cells with different cell diameters measured in junctional/compound nevi (a) and intradermal nevi (b) for each lesion. Case numbers correspond to those listed in Table 1

The nevomelanocytes were significantly smaller (*p* = .0001) in junctional and compound nevi (8.94 ± 2.9 μm) compared to the nevomelanocytes in the intradermal nevi (14.08 ± 3.3 μm). Figure 4 shows the distribution of the nevomelanocyte diameters for the two groups of nevi for different patients.

#### 3.1.3 Percentage of nevomelanocytes outside the nests

Most of the nests (76%) were rather small including less than 45 nevomelanocytes. The nests with the largest number of nevomelanocytes (100–125) were located in intradermal nevi. In most nevi (72%), the nevomelanocytes were located exclusively inside the nests. The rest of nevi showed nevomelanocytes located both within and outside nests. The number of sparse melanocytes outside nests represented less than 5% of those located within the nests.

Elongated rete ridges represented another specific feature we imaged in nevi. We identified this feature exclusively in junctional nevi. We compared the elongation of the rete ridges based on their morphology in the en‐face images. At the dermo‐epidermal junction, the en‐face MPM images show a horizontal sectional view of the rete ridges. In this view, the rete ridges appear as ring‐like structures of basal keratinocytes surrounding the dermal papilla. In the nevi with long rete ridges, this morphology was visualized as deep as ~ 150 μm, the common depth limit for MPM imaging of nevi. For the nevi with shorter rete ridges, the en‐face images captured at ~ 150 μm depth showed the presence of collagen and elastin fibers, that is, the morphology characteristic to the skin dermis. Representative images that illustrate the assessment of rete ridges are included in the Supporting Information, in Figure S4. We identified elongated rete ridges in 6 out of 11 junctional nevi. Nests of nevomelanocytes were not visualized in 5 of these nevi, likely due to their deeper location at the tips of the rete ridges.

### 3.2 Immune response

We captured the immune response in the form of dermal infiltration of inflammatory cells and melanophages. The presence or absence of the immune response was consistent in all the stacks acquired in 80% of the patients. In the rest of the patients, the inflammation was captured on average in 53% of the total number of stacks acquired.

**Figure 5:**
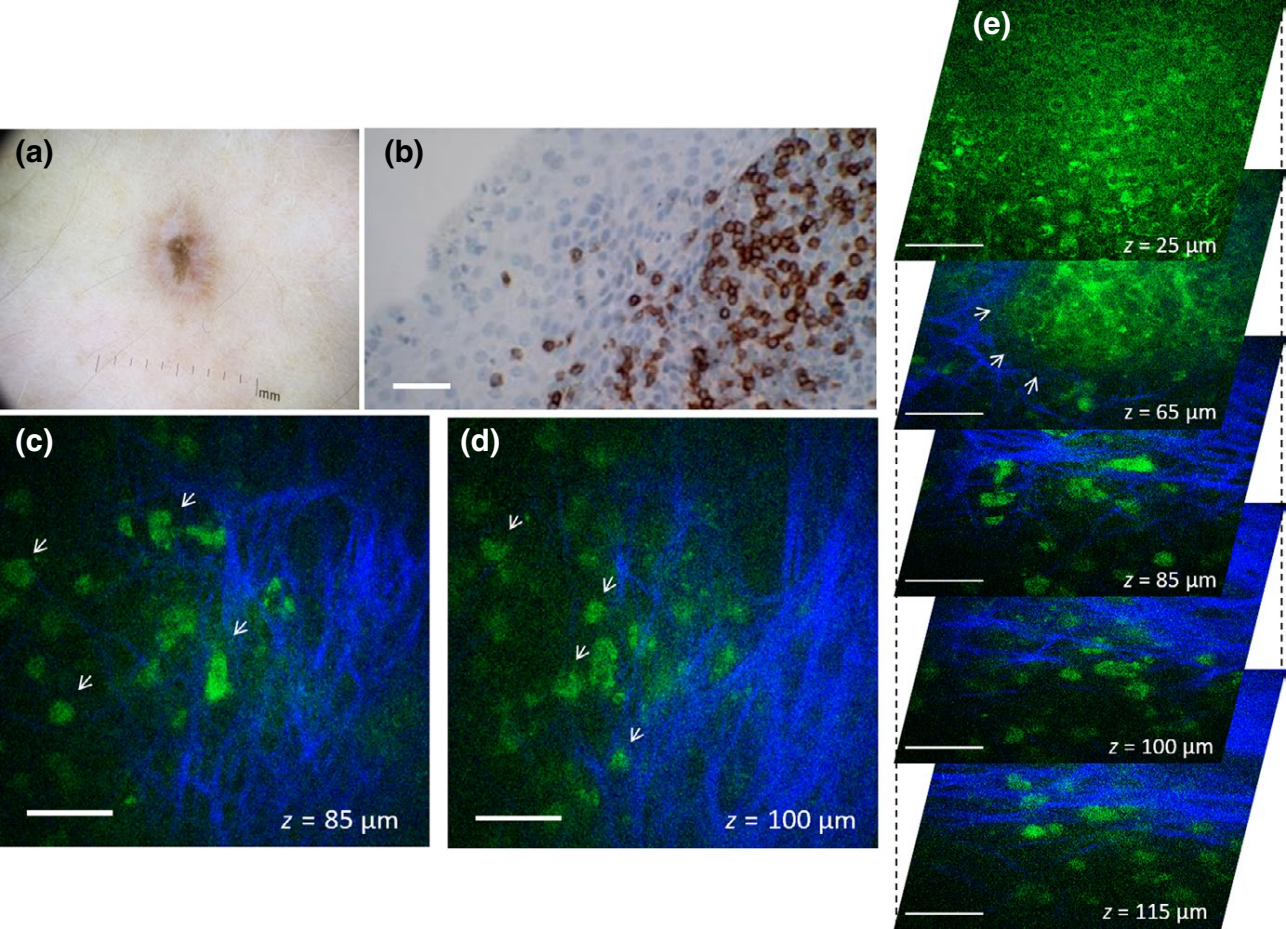
In vivo MPM of lymphocytes in a recurrent compound nevus. (a) Clinical image (Dermlite FOTO, Dermlite, Inc), Case 27. (b) Immunohistochemistry image showing CD3‐positive T‐cell lymphocytes in the superficial dermis; (c, d) En‐face MPM images of the nevus in (b) showing inflammatory cells (arrows) in the superficial dermis at a depth of 85 (c) and 100 μm (d). (e) A stack of en‐face MPM images acquired at different depths from the nevus in (b) showing a nest of nevomelanocytes at the dermo‐epidermal junction (arrows) and inflammatory cells in the superficial dermis. Scale bar for in all images is 40 μm

Multiphoton microscopy imaging revealed the presence of inflammatory cells in two of the nevi imaged in vivo by MPM. These nevi were histologically diagnosed, one as residual dermal melanocytic nevus (Case 20), the other one as recurrent compound melanocytic nevus (Case 27). Figure 5 (b–e) shows dermal cells visualized by MPM in vivo in the recurrent compound melanocytic nevus (Case 27), along with a nest of nevomelanocytes at the dermo‐epidermal junction. The appearance of dermal cells is different from the nevomelanocytes imaged in this lesion or in others (Figures 1, 2, S2, Video S1, Video S2), and based on correlation with immunohistochemistry (Figure 5b), they are most likely CD3 T cells. In the MPM images, these cells were approximately round in shape, were about 10–12 μm in size, and arranged in clusters. Unlike nevomelanocytes, they appear dispersed rather than closely packed and their nuclei are not well resolved.

**Figure 6:**
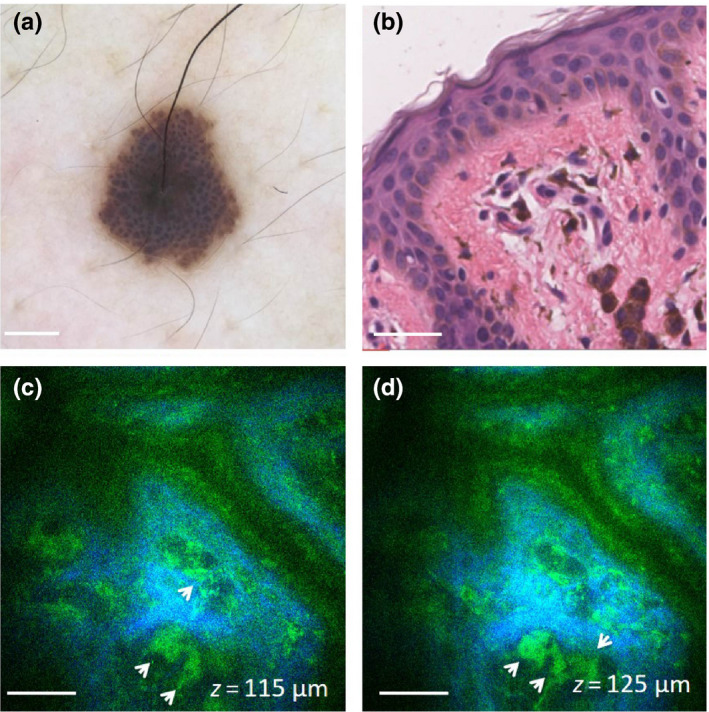
In vivo MPM of melanophages in an intradermal nevus. (a) Clinical image (Dermlite FOTO, Dermlite, Inc), Case 23. (b) H&E histological section of the lesion in (a) showing nests of melanophages in the papillary dermis; scale bar is 40 μm. (c,d) En‐face MPM images of the nevus in (a) showing melanophages (arrows) in the papillary dermis at a depth of 115 μm (c) and 125 μm (d). Scale bar is 40 μm

Multiphoton microscopy imaging also revealed the presence of melanophages in the dermis of eight nevi. Six of these nevi were biopsied and histologically diagnosed as compound (2) and intradermal (4) nevi. In the MPM images, melanophages had an irregular shape, were about 18–20 μm in size, and were generally bright due to fluorescence from their abundant melanin content. They were arranged in clusters, and some of them appeared dendritic. Their appearance correlated well with the melanophages identified in the histological images. Representative example of MPM images showing melanophages imaged in an intradermal nevus is shown in Figure 6 (c,d, Case 23). The presence of melanophages in this lesion was confirmed by histology (Figure 6b).

Establishing the cytological morphology of nevomelanocytes and immune response by comparison with histopathology allowed us to accurately identify these cells in nevi that were not biopsied. Representative MPM images of nevomelanocytes and melanophages imaged in such nevi are shown in Figures S2 and S3, respectively (Supporting Information).

## 4 DISCUSSION

In this study, we assessed both qualitatively and quantitatively the cytological and morphological features of common nevi in human skin as visualized by in vivo MPM.

Nests of nevomelanocytes represented the most predominant feature captured by MPM in most nevi. We assessed several quantitative parameters related to the size of nevomelanocytes, the size of the nests, and the percentage of nevomelanocytes located outside the nests. The nevomelanocytes of intradermal nevi were significantly larger and presented larger nuclei than the nevus cells in junctional and compound nevi. Their appearance correlated well with the corresponding histological images and, based on their large size and epithelioid shape, seemed to represent the superficial component (type A melanocytes) of so‐called “maturation” (Massi & LeBoit, 2013). This term is commonly used in histology to indicate the progression of nevomelanocytes from large epithelioid cells in the papillary dermis to smaller, lymphocyte‐like cells in the superficial dermis and spindle Schwann‐like cells deeper in the dermis. In all nevi, the size of the vast majority of the nests (~90%) was in the range of 20–80 μm and did not significantly change with the imaging depth. The nevomelanocyte nest size can be a potential relevant metric in differential diagnosis of benign and malignant melanocytic nevi. (Pellacani, Cesinaro, & Seidenari, 2005) The limited imaging depth of MPM in skin (~200 μm) did not allow us to measure a potential change in size of the nevomelanocyte nests with depth, but we were able to show that the majority of melanocytes were located within the nests. Published studies have suggested that melanocytes within nests express the senescence marker p16 (Koh & Cassarino, 2018). This observation indicates that the metric related to percentage of nevomelanocytes outside the nests may be used to assess the nevi in evolution. Metrics based on size of nests and/or the percentage of cells located outside nests may also be used as criteria to distinguish nevi from melanoma. This hypothesis is based on observations from histology (Price, Rywlin, & Ackerman, 1976) and from our previously published work on MPM imaging of nevi and melanoma (Balu et al., 2014), but it needs to be tested in further studies.

Elongated rete ridges, a known histological feature of junctional nevi (Massi & LeBoit, 2013), were imaged by MPM in about half of these types of nevi. They generally hindered the imaging of nevomelanocytes nests. Thus, while nests of nevomelanocytes were imaged in most of the nevi, they were not captured in most nevi that showed elongated rete ridges. This was likely due to the location of the nevomelanocytes at the tips of the rete ridges (Massi & LeBoit, 2013) that were too deep to access by MPM. Limited penetration depth of MPM was also likely the reason we did not capture the nevomelanocytes in three out of eight nevi diagnosed as predominantly intradermal.

Inflammatory infiltrates may develop in some types of melanocytic lesions such as recurrent and residual melanocytic nevi that involve regression (Fox, Reed, & Shea, 2011). MPM was able to capture dermal cells with a distinct morphology in a regressing nevus in approximately the same location where infiltrating CD3 + cells were observed on histology. T‐cell infiltration in nevi is often a sign of regression or of an anti‐melanocyte immune response and can be indicative of a good response to immunotherapy (Linck, Costa, & Garicochea, 2017). Although these observation suggest that MPM can definitively identify T cells surrounding nevi based on their distinct morphology, more extensive imaging of skin lesions with characteristic T‐cell infiltrates such as cutaneous T‐cell lymphoma (CTCL) is required to validate this imaging signature. Melanophages, commonly visualized histologically in common nevi (Massi & LeBoit, 2013), were imaged by MPM in the papillary and superficial dermis of two junctional, one compound, and four intradermal nevi. We distinguished the melanophages in the MPM images of the intradermal nevi by comparing their cellular morphology with the melanophages identified in the histological images. Identification of melanophages through comparison of the MPM and histological images allowed us to accurately identify these cells in the junctional nevi that were not biopsied. Similarly, establishing the cytological morphology of nevomelanocytes by comparison with histopathology was valuable in accurately identifying these cells in nevi that were not biopsied. Unlike the nests of nevomelanocytes, dermal melanophages do not represent a specific morphological feature of common nevi. However, they commonly can be indicative of prior inflammatory response in common nevi or regression of melanocytes.

The qualitative and quantitative parameters proposed in this study are instrumental in a future study on an expanded number of lesions in order to evaluate the potential of MPM as a non‐invasive, label‐free imaging tool for diagnosis of pigmented lesions. Such a study would also need to address current technical limitations of the MPM clinical imaging, particularly the reduced scanning area and penetration depth. Improved penetration depth would allow visualization of nevomelanocytes at the tips of elongated rete ridges, immune response of deeper dermis, and the evaluation of maturation within deeper nest. Dispersion compensation to decrease the laser pulse duration (Balu, Saytashev, Hou, Dantus, & Tromberg, 2015; Tang, Krasieva, Chen, Tempea, & Tromberg, 2006) and adaptive optics (Ji, Sato, & Betzig, 2012) are among approaches that have been reported recently for enhancing penetration depth in thick, scattering tissue. Sampling large areas of the lesions are critical to avoid false‐negative diagnoses, as pigmented lesions are often non‐uniform. We have recently proposed an approach for addressing this limitation by designing and developing an MPM imaging prototype device that provides rapid scanning of large skin tissue areas, while maintaining submicron resolution (Balu, Mikami, Hou, Potma, & Tromberg, 2016).

In conclusion, this study illustrates and discusses qualitative and quantitative cytologic and morphologic metrics in common nevi as visualized by MPM. The qualitative descriptors complement the MPM features previously described in melanoma. A large database of MPM descriptors to characterize a broad range of pigmented lesions is critical in the process of discriminating melanoma from look‐alike lesions by in vivo MPM imaging. The quantitative parameters proposed in this study to characterize nevi imaged in vivo by MPM may be extremely relevant in future studies for assessing nevi in evolution or for distinguishing benign nevi from early‐stage melanoma.

## CONFLICT OF INTEREST

K. Koenig is the cofounder of Jenlab, GmbH. M. Balu and B. Tromberg are co‐authors of a patent owned by the University of California, which is related to the technology described in this study. The Institutional Review Board and Conflict of Interest Office of the University of California, Irvine, have reviewed patent disclosures and did not find any concerns. No potential conflicts of interest were disclosed by the other authors.

## Supporting information

Fig S1

Fig S2

Fig S3

Fig S4

Video S1

Video S2

Supplementary Material
