# Supplementary figures and images for: Non‐invasive optical biopsy by multiphoton microscopy identifies the live morphology of common melanocytic nevi
Source: Pigment Cell Melanoma Res. 2020 Jun 17;33(6):869–77. doi: 10.1111/pcmr.12902 (PMC7687135; doi:10.1111/pcmr.12902)

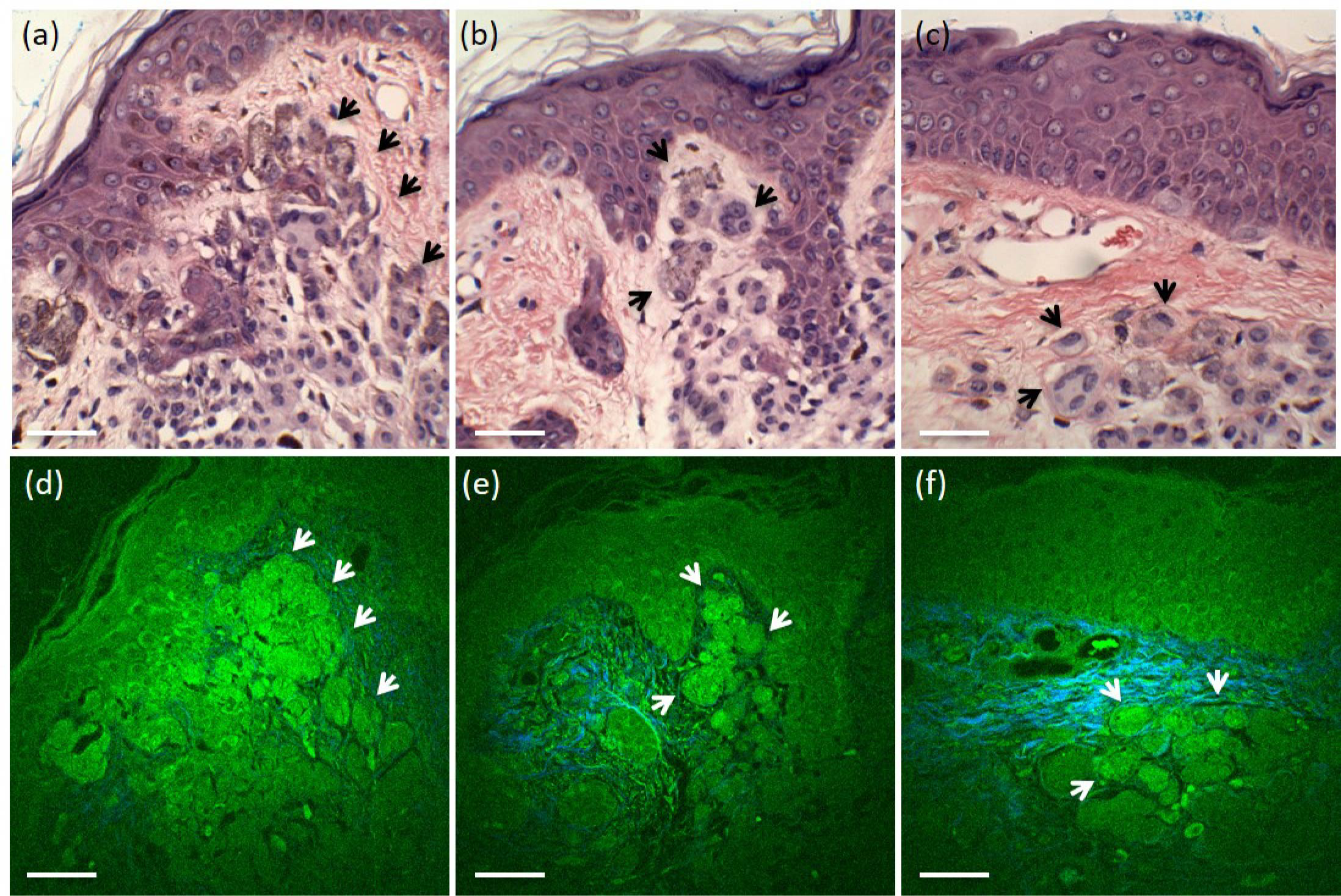

Supplement: Supplementary file 1 — Fig S1 [file PCMR-33-869-s001.png]

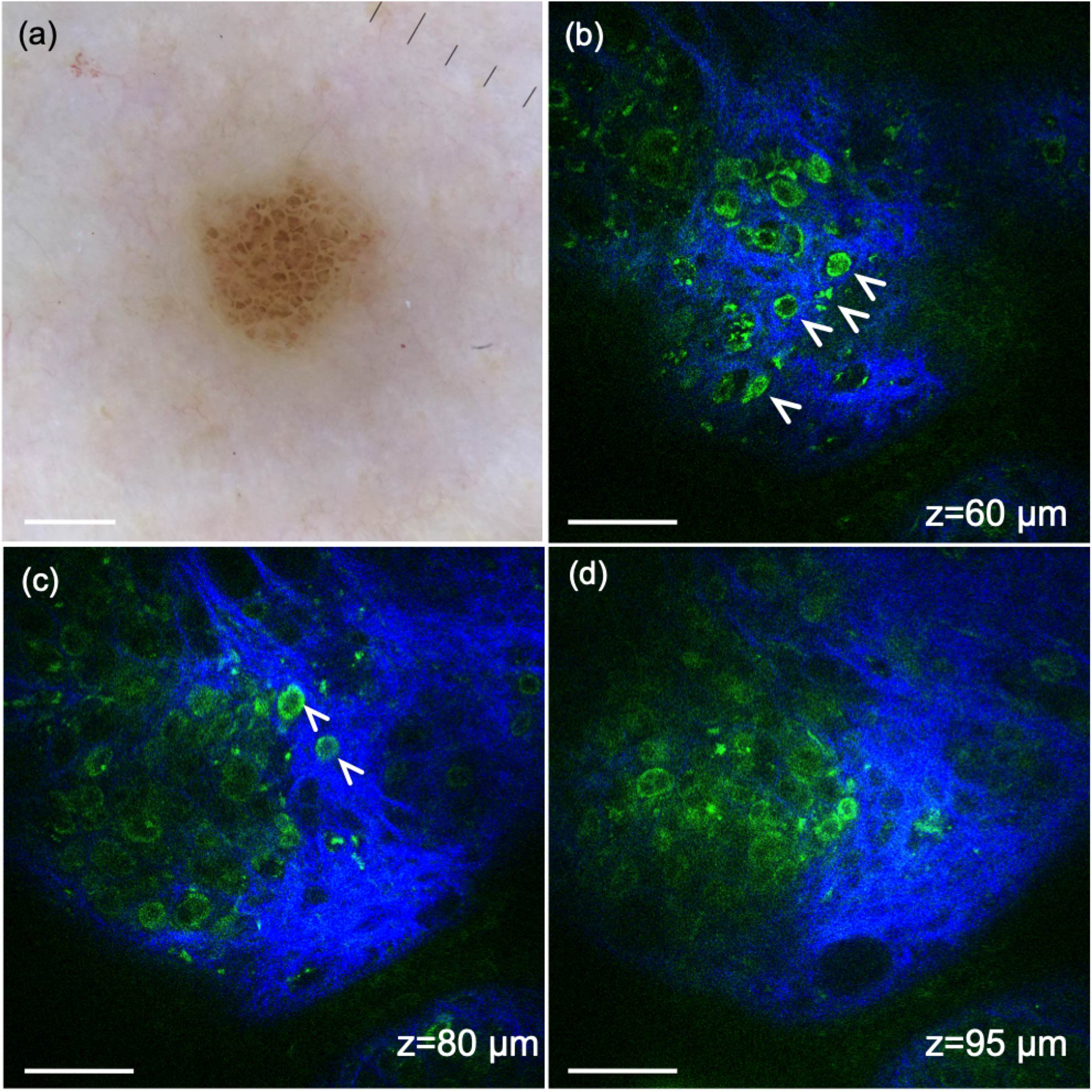

Supplement: Supplementary file 2 — Fig S2 [file PCMR-33-869-s002.png]

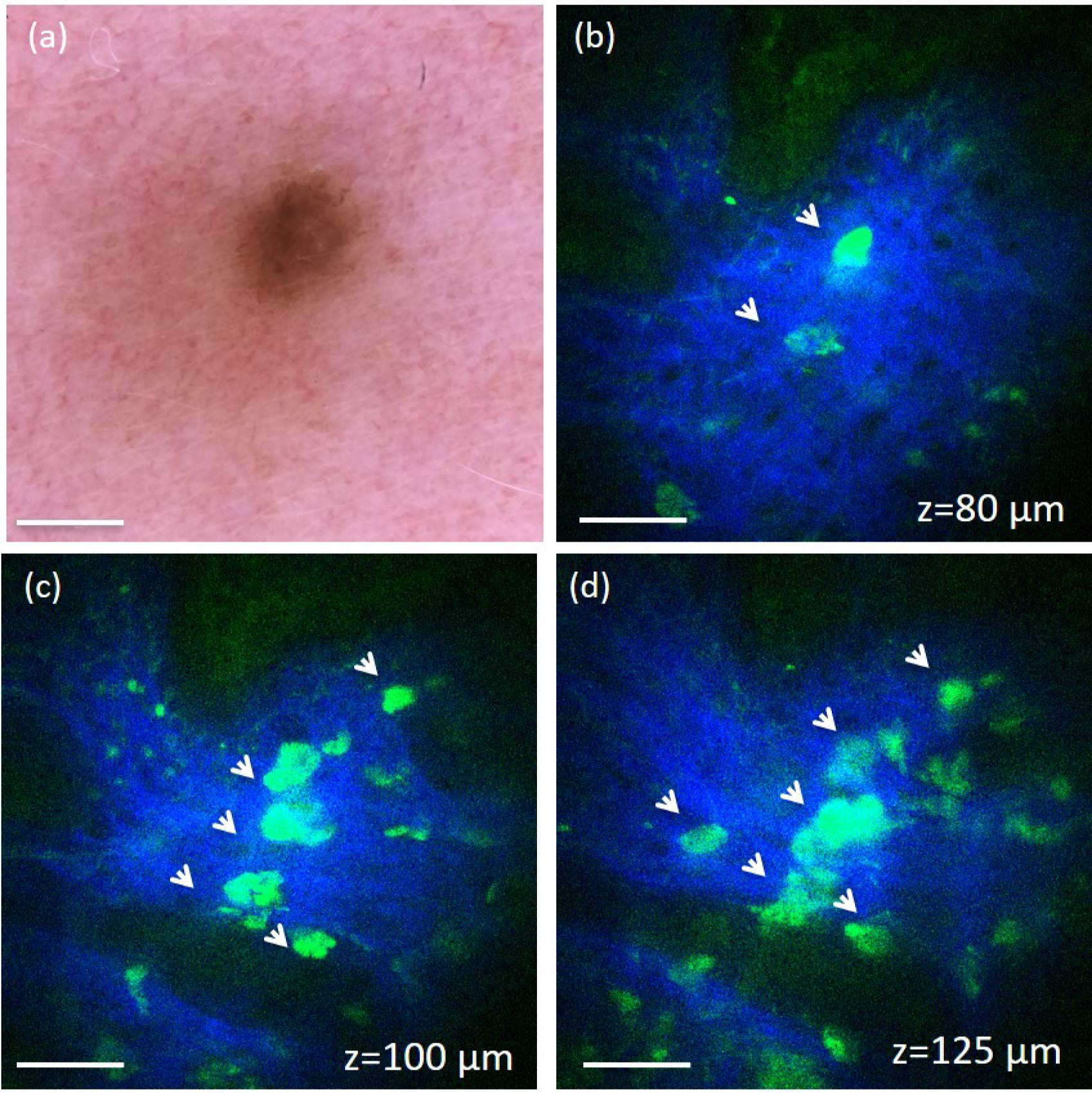

Supplement: Supplementary file 3 — Fig S3 [file PCMR-33-869-s003.png]

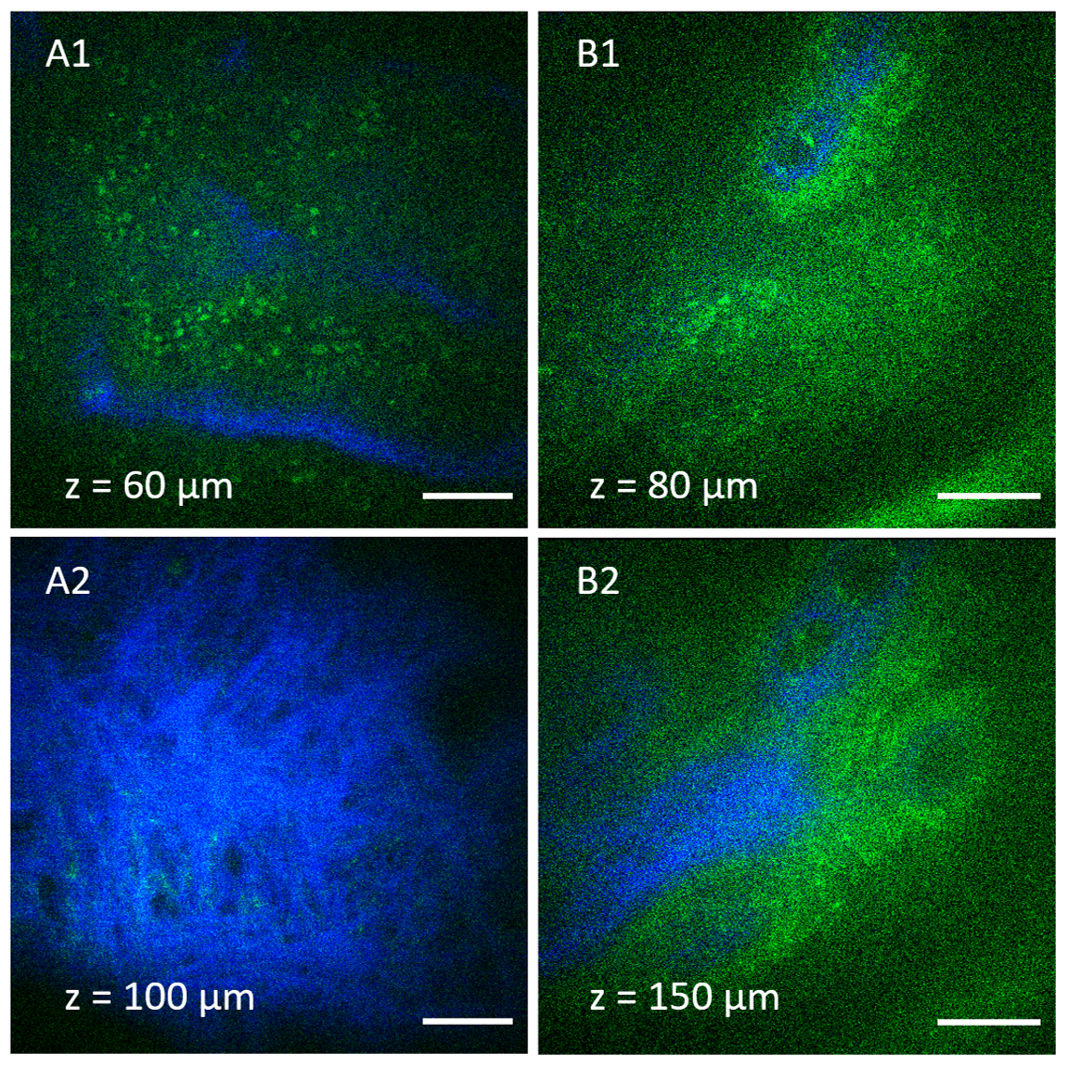

Supplement: Supplementary file 4 — Fig S4 [file PCMR-33-869-s004.png]
