# Supplementary material for: Non‐invasive optical biopsy by multiphoton microscopy identifies the live morphology of common melanocytic nevi
Source: Pigment Cell Melanoma Res. 2020 Jun 17;33(6):869–77. doi: 10.1111/pcmr.12902 (PMC7687135; doi:10.1111/pcmr.12902)
Supplement: Supplementary file 7 — Supplementary Material [file PCMR-33-869-s007.docx]

**Supplemental Figure Legends**

***Figure S1. H&E and MPM images of adjacent tissue sections from an intradermal nevus.*** (a-c) H&E histological sections of the lesion shown in Fig. 2 (Case 25) showing nests of nevomelanocytes in the papillary dermis (arrows); (d-f) MPM images of the successive tissue sections in (a-c) showing the same nevomelanocytes (arrows) in the papillary dermis. Scale bar is 40 μm.

***Figure S2. In vivo MPM of nevomelanocytes in an intradermal nevus.*** (a) Clinical image (Dermlite FOTO, Dermlite, Inc), Case 14 (b-d) En-face MPM images of the nevus in (a) showing nests of epithelioid nevomelanocytes (green) displaying rounded large nuclei and surrounded by collagen (blue) in the papillary and superficial dermis at depths of 60 μm (b), 80 μm (c) and 95 μm (d). Arrows indicate sparse nevomelanocytes located outside the nest. Scale bar is 40 μm.

***Figure S3. In vivo MPM of melanophages in a junctional nevus.*** (a) Clinical image (Dermlite FOTO, Dermlite, Inc), Case 5. Scale bar is 2 mm; (b-d) En-face MPM images of the nevus in (a) showing melanophages (arrows) in the papillary and superficial dermis at a depth of 80 μm (b), 100 μm (c) and 125 μm (d). Scale bar is 40 μm.

***Figure S4. Assessment of rete ridges in nevi based on en-face in-vivo MPM images*.** (A) MPM images of a nevus (Case 17) at a depth of 60 μm below the skin surface showing basal keratinocytes (green) surrounding dermal papilla (blue) (A1) and at 100 μm depth in the superficial dermis showing collagen (blue) and elastin (green) fibers (A2). (B) MPM images of a nevus (Case 13) at a depth of 80 μm below the skin surface showing basal keratinocytes surrounding dermal papilla (blue) (B1) and at 150 μm depth still showing the morphology of the rete ridges in the en-face view (basal keratinocytes surrounding dermal papilla), a sign of elongated dermo-epidermal junction and thus, of the rete ridges (B2). Scale bar is 40 μm.
